# Book Reviews

**Published:** 1811-07

**Authors:** 


### CRITICAL ANALYSIS
### OF
### RECENT PUBLICATIONS
### IN THE
### DIFFERENT BRANCHES OF PHYSIC, SURGERY, AND MEDICAL PHILOSOPHY.

---

*A Letter on the Study of Medicine, and on the Medical Character, addressed to a Student, by* Peter Reid, M. D. 8vo. pp. 59. Murray.

From the title of this letter, we were led to anticipate certain directions which might assist the student in his career of medical attainment. If we have been disappointed in this expectation, we have been abundantly gratified with the strong sense, keen observation, and intrepid remarks of this intelligent writer. But his opinions will probably be approved by the smaller portion of his brethren, and few in the class of students, to whom they are especially addressed, will be able to appreciate their truth, or acknowledge their justice. Before he can admit the due application of the satire, the tyro must have experienced many of the errors of education, and seen many of the vices of practitioners, at which this author aims his shafts.

The following is a fair specimen of that part of the letter, which more particularly concerns those to whom it is addressed.

"In order that you may reap due advantage from your studies, it is of the utmost importance that you should be able to form a just notion of the comparative value of the numerous objects which are about to be presented to your notice, otherwise you may waste that time in frivolous and secondary pursuits, which should be consecrated to solid attainments; for nothing is more common than to see a man, who thinks himself a zealous votary of true science, when he is only busied with its excrescences, or playing with the trumpery at its threshold. It is therefore essential to your progress, that you should be enabled to separate that kind of knowledge which is merely curious from that which best serves serves the purposes of practical wisdom, so that you may not sacrifice the one to the other. Perhaps no other profession requires a stricter guard against those manifold absurdities by which an unsound head is held up to the ridicule of a laughing world. It is one of the unfortunate circumstances of medicine, that foolishness has so many ways of displaying itself; it is so ready to thrust itself upon us, that it requires more than usual circumspection to prevent a man from being hooked up by some of the baits which are hanging out on purpose to seduce him from common sense. Thus if we take a survey of all those men who rank themselves amongst the sons of science, without any fair passport, how many shall we see blowing bubbles with as important an inflation of their cheeks, as if they were making worlds! How many, with their heads stuck with straws, strutting with as much mock majesty as if they had covered them with diadems! In this list we must include all mere collectors of shells and stones, gatherers of weeds, vermin-hunters, makers of random experiments, all dilettanti members of learned societies, gossipping narrators of strange facts, men that have seen wonderful monsters in their day, and have been exposed to many a moving incident by flood and field; all men that stalk about the earth as great observers, solemnly busy in catching butterflies; all those who think that enriching their cabinets with toys is filling their heads with sense; in short all those who resolve to run, jump, stare, dive, cut, dress, sing, fiddle, or dance themselves into men of science."

Dr. Reid proceeds to expose the folly of imbibing the opinions of others till we have none of our own; of mistaking and perverting the purposes of philosophy; playing with paradoxes and spinning cobwebs, &c. He then delineates the character of mind which fits a man for cultivating science with success.

His remarks on the nature of study give evidence of a correct and broad understanding; amongst several which we might quote with approbation, the following ought to be generally known, and impressed on the mind of every student.

"There are many who think themselves entitled to the claims of a liberal education, from having read a great many books, and punctually attended all the classes, without ever considering that, while the mind remains passive, nothing valuable can be attained. They therefore often find, when it is too late, that going over a certain quantity of print with their eyes, hearing lectures, and seeing cases, have imposed on them with a specious shew of activity; that all these are only the means of collecting the materials of knowledge, which, without being weighed and sifted by their judgment, only seem to oppress the memory, without enlightening the understanding."

Dr. Reid cautions young men against indulging in the luxury of light reading, and warns them not to suffer the pursuit of polite literature to encroach too much on the time which should be employed in professional avocations. This reading also, he observes,

"Has often a very amusing effect on certain heads; it invites them to to lighten up their scientific performances with very pretty poetic effusions; they acquire that dashing figurative style of writing, which hits the happy medium between poetry and prose, vulgarly called bombast, and is so sublimely bad, that it is much better than if it was good. It is not every one that can steer

"From grave to gay, from lively to severe,"

and still keep up that due balance between the fancy and judgment, which prevents the operations of the one from encroaching on the provinces of another."

The student is very properly cautioned not to suffer any interesting collateral branch of medical science to allure him into two close a pursuit of it, to the exclusion of those with which he must also be acquainted: as the ability and the usefulness of the physician depend not on his being a great anatomist, chemist, or physiologist.

The author's remarks on attending lectures are very acute.

"There are many students (he says) who deceive themselves most egregiously in their attendance on lectures. They are occupied in a continued round of classes, where one object thrusts out another in endless succession, and a medley of demonstrations, experiments, facts, observations, theories, and hypotheses, this man's opinion, and that man's opinion, fleet through the mind without leaving any trace behind, or breed such an uproar in the understanding, as confounds all judgement and reflection; just as if the mind was to be valued, like a sieve, not for what it retained, but for what passed through it. Hence, when they find that it is impossible to retain one-hundredth part of what they hear, or to meditate upon and digest one-hundredth part of what they retain, they devolve the duties of the head on the fingers, and think, that as they fill their note-books, they are improving their understanding."

In illustration of this author's talent for ridicule and power of caricaturing, we might quote several pages in which he considers the various ways in which practitioners attempt to acquire business, but our limits will only permit us to insert the following by way of sample.

"There is a particular bustling gait and hurried step, somewhat resembling that of a barber in a morning, which is thought to imply, that a man is harassed to death with business, which, according to the proverb, is thought the best security for more. As I have seldom any calls that require a very hurried pace, being often at perfect liberty to saunter wherever my fancy leads me, I have sometimes amused myself with keeping a sharp look-out on the motions of these worthies, and have seen occasionally a corner turned with a dexterity that would have done credit to any jockey; and once had the curiosity to overlook a long paper, meant for a list of patients, which one of these men of bustle was perusing in the streets with a serious face; and he had good reason, for a more formidable tailor's bill I never saw. In many places a chariot is a most indispensable vehicle for conducting a man into practice; and indeed I do not know any sight more interesting than the full profile profile of a sapient physician, so judiciously fixed, as to be seen with equal advantage from both sides of his chariot, while he himself appears totally unconscious of the vulgar gaze, and enjoys the supreme pleasure of putting a poor pedestrian brother to flight, by the jehu whirl of his triumphal car."

"If we wish to amuse ourselves with higher game, we have the medical coxcomb, who moves only in the higher circle of fashion, is himself ambitious of being thought a fashionable man, and can only cure people of quality, like the knight of La Mancha's balsam, which was only good for those of the same order. This creature of grimace, in straining after that elegance of manners, which is so engaging when it is the expression of real delicacy of sentiment, for want of this necessary fineness of perception, works itself into the most grotesque motions imaginable, and becomes a most valuable specimen of the ridiculous, very useful in pointing out to children the hideous effects of affectation. Come forward, then, thou hollow, heartless, brainless thing, and let us examine whether thou art a man or an ape: ah! it smiles, it dances, it sings; its notes swell highest at the concerto, it displays the elegance of a figurante in the dance."

But it is time to conclude, we have quoted enough to elucidate our author's varied powers of writing. We regret that so many pages of this small treatise are devoted to caricatures, the drawing of which, in our opinion, is so incorrect and overcoloured, that the objects intended to be pourtrayed cannot be recognised. Surely the author would have conferred more essential benefit on his young readers, by more minutely pointing out the difficulties which impede their acquisition of principles which are to form the basis of their medical education: Surely, in addressing himself to medical students in particular, he might have spared himself the labour of embodying in language, many of the tasteless conceptions which obtrude themselves on our notice in various parts of his work. Alluding to the facility which the uncertainty of our art affords for sporting with popular credulity, he observes,

"Indeed, many have a strong suspicion that medicine is one of the black arts, and that we work by witchcraft; hence many find their account in pretending to supernatural gifts; and we are told that Paracelsus *Dæmones præceptores habuit, Dæmones familiares*. It is upon this principle that I strongly suspect that several very great conjurors in our art have *sold their souls* to the devil; this, however, I could by no means venture to affirm positively, as it is only from some of their slights of hand that I should suspect *they had compounded for a roasting hereafter*." P. 43.

In a second edition, we trust that Dr. Reid will have the courage to omit some of these *witty* passages, and that he will considerably extend the useful parts of his letter, for which he has proved himself eminently qualified.

*Essay on some of the Stages of the Operation of Cutting for the Stone. Illustrated with an Engraving.* By CHARLES BRANDON TRYE, F.R.S. 8vo. London, 1811. pp. 49. Callow.

THE objections which have lately been urged against the employment of the *Gorget* in Lythotomy, and the recommendation of the *Scalpel* for dividing the integuments, muscles, urethra, and prostate, have induced Mr. Trye to become the advocate of the former instrument. The objections to the *Gorget* are distinctly stated from Mr. Allan's publication; and to each objection Mr. Trye annexes an answer.

" Obj. 1. ' The beak of the gorget is apt to slip from the groove of the staff, before it reaches the neck of the bladder. This has often happened in the hands of good operators, and assuredly will happen.'

" Answ. If this happens, the staff and beak of the gorget must be both very ill made, or the surgeon must enter the urethra too soon, for instance, in the bulbous portion; whereas, he ought never to pierce that canal but in the membranous portion, and within half an inch of the prostate gland; and as then the gorget will not have more than the third of an inch to traverse, before it comes to the prostate, it will scarcely lose its way in this distance, and in the hands of a good surgeon the handle of the gorget is always depressed, in proportion as he depresses the handle of the staff.

" Obj. 2. ' If the operator presses his gorget onwards too horizontally, the prostate gland, being moveable, will recede, the gorget slip from the groove, and be driven between the rectum and the bladder.'

" Answ. It is here assumed that the surgeon operates with a very dull edge to his gorget; whereas it should always makes its way as easily as a scalpel would do; as will be the case, if its shape be good and its edge keen and smooth."

" Obj. 3. ' If the surgeon, or assistant, depresses the handle of the staff too much over the right groin, with the idea of making its bend or heel be distinctly felt in the left side of the perineum, the point of the staff will slip out of the bladder, and when the surgeon has completed his external incisions, it will start through the membranous part of the urethra; and, in this case, pushing his gorget by this false guide, he will drive it between the bladder and rectum.'

" Answ. If the staff be started through the membranous part of the urethra, it will also misguide the knife of the operator, so that this is no specific objection to the gorget, but only to an aukward surgeon, using an ill contrived staff, and having an aukward assistant. However, I hope I have freed the staff from being liable to its share of this censure.

" Obj. 4. ' It is uniformly acknowledged by the best surgeons, that the gorget cuts the prostate gland very imperfectly. Its incision sometimes admits, with difficulty, the introduction of the forceps; and if the stone be large, is quite inadequate to its extraction, without dreadful laceration.'

" Answ.

"Answ. If the gorget does not divide the prostate sufficiently, the fault is in its make, not in the principle of using it. It is not necessary nor expedient that the wound, through which the stone is to pass from the bladder, should be of the same length as the longest diameter of the stone.

Sir James Earl has given plates of the very large stones which he has successfully extracted after using the gorget; and larger than those will rarely occur to any operator: and after all, if the surgeon finds he has a stone too large to be extracted without dreadful laceration, he can enlarge the wound of his gorget by his scalpel, just as well as if he had never used the gorget at all; for we cannot suppose, that a prudent surgeon, even if he confines himself to the knife, will make his incision with his knife of the greatest possible size, before he has ascertained that the stone is of an extraordinary magnitude; which he cannot certainly do, till he has got his forceps and finger into the bladder. Neither the staff, nor feeling through the medium of the rectum, will enable him to ascertain the size of the stone.

"Obj. 5. 'If the cutting part of the instrument be made broad, to provide against the last accident, it enters the pubis with great difficulty, grates the bone, by which the pubic artery is sure to be wounded, and the patient brought into great danger by the hæmorrhage, which commonly proves fatal.'

"Answ. I admit the objection; but I should be sorry to see any surgeon operate with a gorget, which justified the expectation of its doing so much mischief.

"Obj. 6. 'Whenever the gorget enters the bladder, the patient feels an irresistible inclination to bear downwards, by which the fundus of the bladder is pressed against the point of the instrument; and if it be kept long in the bladder, to serve as a conductor to the forceps, this generally happens.'

"Answ. I never felt this descent of the fundus in using the prostatome; and my finger has always been so instantaneously introduced along its channel, that it could not have happened without my perceiving it: and, as I never keep this instrument long in the bladder, nor use it as a conductor to the forceps, at all events, I obviate in practice this part of the objection, whatever its force may be.

"Obj. 7. 'It is possible for a rash surgeon to push the gorget on with such violence, as to transfix the bladder.'

"Answ. There is no guarding against the mischiefs of rashness, whether a man use the knife or any other invention. However, if a square stop terminate the groove of the staff, as in that which is here delineated, it must be a very rash surgeon indeed, using a very strong hand in a very violent hurry, who, notwithstanding this defence, can pierce the opposite side of the bladder.

"Obj. 8. 'When the gorget has been successfully introduced into the bladder, and all these dangers have been avoided which we enumerated, unless the operator be very careful in withdrawing it in the very position in which it enters, it will make another incision.'

"Answ. This accident is very effectually prevented by me, and, I suppose, by all operators who do not introduce the forceps on the gorget, by withdrawing it with its cutting edge under the fore finger of the left hand, which certainly keeps the bladder from coming in contact with it, while the instrument is being withdrawn by the right hand.

"Obj. 9. 'No man of feeling ever witnessed the *plunge* of the gorget, when *driven* into the bladder, without horror, or did it without reluctance.'

"Answ. To this appeal to our sensibility I must briefly reply, that the gorget, or whatever instrument be used, should never, in its introduction, excite the idea of *driving* or *plunging*. The operator should carry forwards his instrument as coolly, deliberately, and with as much manifest command of his hand, as he would in bleeding open the basilic vein, with the artery placed immediately under it.

The character, extensive practice, and success of Mr. Trye, give considerable value to his opinions, which are unequivocally in favour of the gorget; the form of which, however, he has altered in some particulars, and has described the altered instrument under the name *prostatome.* A plate renders this description perfectly intelligible, as well as some peculiarities in the staff and forceps which Mr. Trye uses in his operations.

Having thus replied to the objections brought against the gorget, the advantages said to arise from employing the scalpel exclusively are examined.

"Is it probable," Mr. Trye asks, (p. 16.) "that the prostate will be divided with equal precision, as to direction and dimensions, and that the adjacent parts will be equally protected, if we reject every instrument besides the scalpel? I know before hand the size of the wound which the prostatome produces, and by observing the relative position of the staff, in what direction it will be made. For as the handle of the staff is inclined more or less to the right groin of the patient, so will the wound in the prostate deviate, more or less, from a right angle with the wound in the urethra. How many surgeons are there, possessed of such a delicacy of touch as to conclude the work with the knife, with equal exactness? The knife cannot here be directed by the groove of the staff, as it is, in other cases, by the groove of the common director. For the staff, in dividing the prostate, is merely a goal from which the knife must set out, and to which it is to return. During the immediate act of dividing, it must move at some distance from the staff; that is to say, if the prostate be cut by a wound, forming more or less a right angle with the wound through the integuments, muscles, and urethra, which, to ensure the safety of the rectum, must be always done. Mr. Allan's description of the operation by the knife alone, as well as his plate of the lateral incision of the prostate, demonstrate the correctness of my statement. *(See Allan on Lithotomy.)*

"'Tis true that, in the living body, the feel of the prostate is very different from that of every thing beside in its vicinity; and every part of it may be distinguished by an experienced touch. But the finger will rarely have attained that advantage the first time the surgeon is called to cut for the stone; it cannot have acquired it by exercise on the dead body, in which the feel excites a different sensation from what it does applied applied to living parts. Whereas, if a surgeon, having that anatomical knowledge, without which no man deserves the name of a surgeon, is cool and steady, and in the habit of using instruments, and attends to rules, he will, in his first operation,* equally as in subsequent ones, make his way correctly into the bladder. He is guarded against wounding the rectum, vesiculæ seminales, and seminal ducts, all exposed to injury, even from the most skilful hands, provided with the knife alone.

"There are two circumstances which appear very unfavourable to cutting with the knife alone: the patient being a very large and tall man, and the patient being a very small child. I have this day, March 8th, 1809, operated on a very tall man, sixty three years of age, and while I was dividing the urethra, I paid particular attention to the prostate. I am convinced, though I am tolerably accustomed to the use of the knife, and not very deficient in anatomical knowledge, that if I had attempted to have divided the prostate with the knife, I should have certainly been embarrassed by the great depth of the prostate, nor have perfected my task with accuracy.

As, in early childhood, the prostate is too small to be felt, we want its guidance, as to the situation and extent of the incision to be made by the knife. Whereas by our previous consideration of the size, and other circumstances of the prostatome, we can predetermine the situation and dimensions of the finishing wound."

We shall conclude our analysis of this short pamphlet with quoting from it the following singular case of *Ischuria Vesicalis*, subsequent to lithotomy.

"A farmer seventy-five years of age, six feet in height, having been long afflicted, with a stone in the bladder, in other respects in good health, submitted to lithotomy last November.

"The stone was small, and was extracted without difficulty. He was free from unusual pain till the second night, which he passed very uncomfortably, by reason of severe pain about the wound and along the urethra; the following day it was worse, and extended even to the hypogastric region. From circumstances, it was evident that he was free from peritonitis; but that the bladder was suffering from being distended with urine. A female catheter being passed through the wound, did not reach the retained water: a caoutchac catheter was then introduced by the penis, and came out again through the wound: a male silver catheter, in the common way, drew off a large quantity of urine. The same instrument was had recourse to twice or three times a day for a week, and then a caoutchac catheter being introduced with ease, a plug was inserted in its mouth, and withdrawn once in two or three hours, to discharge the urine. Thus went on the second week; after which the catheter was finally withdrawn: then the urine again issued through the wound. In a short time it began again to flow through the penis; in six weeks it all came that way; and he was able to leave Gloucester the seventh week, though the wound was not entirely healed. This is the only instance which I ever knew or heard of in which ischuria vesicalis occurred during the cure of the wound made by lithotomy."

* If he employs the prostatome, we presume the author means.

