# Corrigenda

**Published:** 1811-07

**Authors:** 


					P. 73 and 75, in the running Head, for Dr. Keid on the Study of
Mcdicine, read Mr. Trye on Operations lor the Stone.
Printed, ly E> Hmstcd, Great New Street, Fetter Lane.

				

